# Making the ‘genetic counsellor’ in the UK, 1980–1995

**DOI:** 10.1136/medhum-2022-012472

**Published:** 2023-04-17

**Authors:** Jenny Bangham

**Affiliations:** School of History, Queen Mary University of London, London, UK

**Keywords:** History, reproductive medicine, Genetics

## Abstract

The professional identity of the ‘genetic counsellor’ first took shape in the UK in the early 1990s, when the University of Manchester established the country’s first masters-level training course. Postwar, genetic counselling had been carried out by (male) clinical geneticists, who, alongside their research, clinical and field-building activities, met patients and families to discuss inherited conditions and risk estimates, and who sometimes advised parents whether to attempt or continue pregnancies. By contrast, the new cohort of students in Manchester in the 1990s were not medically trained, were mostly women, and were schooled in the psychological and social consequences of genetic testing and diagnosis, as well as methods for the care, support and emotional management of patients and families. This was a significant change both in the practices of ‘genetic counselling’ and who was expected to practise it. Focusing on a small section of this history, between 1980 and 1995, this paper describes some of the historical threads that contributed to this change. It charts the early work of genetic nurses and social workers, who in the 1980s carved out distinctive roles within National Health Service genetics centres. It describes the separate, specialist provision developed by sickle cell and thalassaemia counsellors, who developed new approaches in dialogue with racialised and underserved patient communities. It examines growing interest in the late 1980s and early 1990s in the tacit social and cultural conditions of genetic counselling encounters, and how this cohered with attention from disability scholars, psychologists and social scientists. By describing these historical contributions, this paper explores how the intersecting gendered, racialised and disciplinary politics of clinical genetics shaped the new professional role of the ‘genetic counsellor’.

## Introduction

‘Genetic counsellors’ are healthcare professionals who help patients and other clients interpret the results of genetic tests and negotiate decisions about reproduction and treatment. Today, clients usually encounter genetic counselling during pregnancy, cancer treatment, or in the course of paediatric care, although increasingly they might be given referrals from other hospital departments.1 Genetic counselling encounters involve the communication of highly technical information; they are also often emotionally charged, and have the potential to impact the lives of individuals, families, extended families and communities. Practices of genetic counselling have a 70-year history in the UK—a history that has been shaped by the disciplinary and gender politics of the National Health Service (NHS), disability rights, racial politics, and ethical and legal debates about reproductive autonomy and genetic technologies. The history of genetic counselling in the UK has, in large part, not yet been written, but it serves as a vantage point for understanding how genetics has been communicated and given meaning within a nationwide health service.2

The identity of ‘the genetic counsellor’ consolidated in the UK in the early 1990s, when the University of Manchester established the country’s first degree-level training course. The course was the culmination of a remarkable shift in genetic counselling practices over several decades. For several decades postwar, genetic counselling in the UK had been carried out by (male) clinical geneticists, who, alongside their clinical, research and field-building activities, met patients and families to discuss inheritance and risk, and who sometimes gave advice about reproductive decisions, before or during pregnancy (eg, Stevenson and Davison 1970; Carter 1971). By contrast, the new Manchester cohort in the 1990s were mostly women, were science graduates or nurses, and were schooled in the psychological and social consequences of genetic testing and diagnosis, as well as methods for the care, support and emotional management of clients and families. This was a significant shift both in the meaning of ‘genetic counselling’, and in who was expected to practice it. How did these changes come about? In this paper I focus on a small section of this history, from 1980 to 1995.

Here I trace in particular the growing significance of care, emotional management and psychotherapeutic interactions in genetic counselling.3 Building on a growing literature on the gendered experiences of professionalisation and visibility in science and medicine (Bangham, Chacko, and Kaplan 2022; Egginton and Thomas 2021; Hicks 2017; Milam and Nye 2015), I draw attention to the professional women who helped to promote these practices, and to their changing roles, identities and visibility within the NHS. In some respects, these developments owed much to earlier changes in the USA, where the first masters-level training course for genetic counsellors was established in 1969 (Stern 2012). In the USA, those newly qualified, female, counsellors emphasised their emotional, caring and counselling expertise to create new niches for themselves within the ecology of medical genetics centres, hospitals and specialty clinics (Stern 2012, 107; Stillwell 2015). One purpose of my paper is to examine why the dynamics of change were so different in the UK. In the NHS—where clinical genetics transformed after 1960 from a tiny, ad hoc medical specialty into patchy, but regionally organised, hospital-based ‘genetics service’—staff worked in teams of clinical geneticists, clerical assistants, laboratory staff, and ‘genetic nurses’ or ‘genetic social workers’ (Harper 2020).4 Section one of this paper follows the work of those nurses and social workers who, in the 1980s, carved out distinctive practices and roles relating to the care and emotional management of clients and families.5

The 1980s was also an important decade for the separate provision given to patients and families affected by the serious and unpredictable heritable haemoglobin conditions sickle cell disease (SCD) and thalassaemia. These conditions have tended to affect people with recent ancestry in Africa, the Mediterranean and South Asia—groups that, in the UK, often also experienced considerable racism and neglect within the postwar welfare system. Ignorance, racism and lack of training within the NHS meant that patients suffering from these conditions experienced inadequate or no healthcare during the postwar decades (Bivins 2015; Bryan, Dadzie, and Scafe 1985).6 As a consequence, a small number of community activists, nurses and doctors pushed for the formation of centres dedicated to the care of people affected by these conditions (Nathoo 2001; Redhead 2019, 2021; Valier and Bivins 2002). Some years before their counterparts in the genetics centres, these clinics established the official title of ‘counsellor’ (sometimes ‘sickle cell counsellor’, or ‘thalassaemia counsellor’); these were nurse health visitors who ran the clinics and developed new roles for supporting patients and their families. Section two of this paper focuses on this specialist provision, and explores how the counselling approaches of sickle cell and thalassaemia clinics were informed by the needs of the UK’s racialised and marginalised populations.

Genetic nurses and social workers, and sickle cell and thalassaemia counsellors, all contributed to a growing interest in the late 1980s in the tacit social and cultural conditions of genetic counselling—the topic of section three. Many practitioners perceived that the social and cultural experiences of clients and professionals could profoundly affect the kinds of information that could be communicated, how that information was understood, and what kind of support was possible. Genetic information did not mean the same thing to all people: the way in which it was assimilated and acted on varied with social, cultural, gendered, ‘disablised’ and ‘racialized’ identities and experiences.7 As the Human Genome Project attracted public attention, these insights chimed with growing interest by social scientists in the varied social and psychological experiences of clients and families faced with clinical genetic information. These themes gained a prominent place in the first UK masters-level training in genetic counselling, which was planned and implemented in Manchester by US-trained genetic counsellor Lauren Kerzin-Storrar. Section four explores how Kerzin-Storrar and her colleagues strategically created a niche for ‘the genetic counsellor’ within the NHS genetics services, in the face of unease from some professionals with a stake in the future of the profession.

Thus, this paper attempts to trace the history of a specific, ephemeral, talking-based, medical encounter.8 It describes how a range of healthcare professionals, and (later) researchers, increasingly envisaged, represented, described, theorised and practised genetic counselling encounters, and argues that they, in effect, ‘made’ or indeed ‘reinvented’ genetic counselling as a psychosocial, emotionally charged interaction.9 To tell this history I use published sources and archives, including papers in the possession of the people I write about. I also rely on a series of interviews conducted during 2021 and 2022 with former counsellors and nurses.10 Writing this during an early stage of my project, the people whose testimonies I use here have tended to be those who stayed within the orbit of genetic counselling throughout their careers, and who had significant roles in shaping the trajectory of its practices. Therefore, the people and places I focus on were usually at the vanguard of change; in other units and centres, change was sometimes slower, or implemented differently, and I will cover those important stories elsewhere. Moreover, in inviting people to participate in interviews, I asked them to ‘look back’ to the 1980s after sometimes 30 years within the still-changing profession of clinical genetics; their later experiences may affect the language and concepts that those interviewees used to describe those early years. Thus, throughout this paper I have attempted to make these orientations clear.

### ‘A Developing Role’

What roles did genetic nurses and genetic social workers have in the NHS? By 1980, clinical genetics in the NHS had been expanding for three decades, and had grown from a few ad hoc units run by clinicians with special interests in genetics, to a total of 16 genetic centres across the ‘regions’ of the UK, led by trained clinical geneticists (Coventry and Pickstone 1999; Harper 2020). A key moment in the expansion and reframing of clinical genetics had been the regional reorganisation of the NHS in 1973, in advance of which some clinical geneticists had pushed for greater resources (Webster 2002, chapter 2). Clinicians consulted prior to the NHS Reorganisation Act argued that the adoption and expansion of prenatal testing, and the change in the UK abortion law in 1967, had put pressure on existing services. By the early 1980s, regional provision was still uneven, but the more established genetic services operated in teams of consultant and training clinical geneticists, laboratory staff, administrative staff, and one or more nurses, health visitors or social workers.11 The latter were sometimes referred to in the 1980s as ‘auxiliary’, or ‘non-medical’ health workers (so-called because they were not trained doctors).

Who were these ‘auxiliary’ workers and what did they do? The earliest such appointment was apparently made in 1959 by Cedric Carter, who ran genetics clinics at Great Ormond Street Hospital and a Medical Research Council unit at the Institute of Child Health. Carter recruited Kathleen Evans, a medical social worker at the hospital, who prepared information on clients and their families prior to clinics, who talked to the families before they met with Carter, and who accompanied Carter in the clinics (Reynolds and Tansey 2010, 29).12 One geneticist later recalled that Evans’s role was to soften Carter’s ‘rather perfunctory style’; he remembered Evans ‘rush[ing] out and comfort[ing] somebody in the corridor because they were visibly upset’ (Reynolds and Tansey 2010, 30). Colleagues recalled Evan’s role in the clinic as ‘informal’, a feature that may have been understood as crucial to her work (Egginton and Thomas 2021), and we have few clues as to the day-to-day encounters she had with clients and families.

We know more about the responsibilities of the first so-called ‘genetics field worker’ in the UK, a woman named Edith Quinn who was a senior registered nurse when appointed in 1971 by clinical geneticist Rodney Harris to work in the Manchester Department of Medical Genetics. Two years later, he and Quinn coauthored a memo explaining her multiple responsibilities in the department’s research and clinical work (Harris and Quinn 1973). Like Evans, Quinn prepared the ground ahead of clinics, obtained hospital notes and other records, met clients and relatives, and constructed preliminary family trees. She was present at clinics when Harris met families to take a detailed medical history and make examinations, and she would order chromosome and other tests. Harris and Quinn used the phrase ‘field work’ apparently to refer to the journeys that Quinn made outside the confines of the clinical setting—her trips to family homes and to the inpatient and outpatient clinics in local hospitals to collect information and blood specimens.13 In making a case for a more widespread role for ‘genetic field workers’ in NHS genetic centres, Harris commented: ‘it is not possible to carry out effective genetic counselling without the services of a trained field worker such as Mrs. Quinn’ (Harris and Quinn 1973).

Others evidently agreed with Harris, and over the next decade, centres around the country brought in nurses and social workers to take on similar roles as those of Evans and Quinn. As their work gradually began to be acknowledged in print, colleagues described the expertise of nurses and social workers in gauging the attitudes and responses of families, and offering appropriate support. In the textbook *Practical Genetic Counselling* (1981), Cardiff-based clinical geneticist Peter Harper praised the important work by ‘auxiliary workers’ in clinical genetics, noting that they were uniquely able to ‘detect problems that the family have not spoken about, and ensure that they have actually understood what the person giving genetic counselling thinks they have’. But Harper saw these activities as distinct from ‘genetic counselling’, which was the preserve of clinical geneticists.14 In Harper’s view, ‘counselling’ was inseparable from diagnosis and the communication of risk estimates (Harper 1981, 116).

Harper was not alone. In services across the country, nurses performed supporting roles to clinical geneticists, who had authority over clinics, and made decisions regarding the information that needed to be collected from which clients and families.15 But by the mid-1980s, this was beginning to change, although in different ways and at varied paces in different places. At the Nottingham service, for example, genetic nurses had previously tended only to meet families in clinics and during follow-ups, but in the mid-1980s, they formally changed the sequence of encounters experienced by families, so that genetic nurses could establish relationships prior to clinical appointments (Guilbert 2021). Such meetings, at the hospital or in the home, were partly to gather family information—such as family tree, blood for tests, or family photographs—but also functioned to help the genetics team understand how a family might respond to the information they were being given. As genetic nurse Penny Guilbert put it, ‘[we would] get [an] impression of what sort of value base they were working from, how did that family tick and how could the information that we might be sharing with them be integrated into that family setup’ (Guilbert 2021).

During the 1980s, published accounts began alluding to the ‘psychological’ aspects of nurses’ work. A Clinical Genetics Society report published in 1982 explained that nursing staff hired to the genetics services should have experience in community medicine, paediatrics and obstetrics. People with this training, it explained, ‘will be aware of the many problems resulting from genetic disease in families, and should be sensitive to the psychological implications for the affected individuals and their relatives’ (Fitzsimmons et al. 1982, 11). Looking back, genetic nurses reflected extensively on the value of psychological support that they gave to families. ‘To expect [families] to come through just to a traditional outpatient appointment with nothing in the terms of care, so to speak, is probably not going to help them to integrate or adjust … so it was about facilitating that adjustment process’ (Guilbert 2021).

The growing visibility of these practices was driven partly by a new forum that genetic nurses and social workers created in the late 1970s for discussing and comparing practices with professional peers. In 1980, they formalised their meetings by establishing the Genetic Nurses and Social Workers Association (GNSWA, pronounced *g-nas-wa*). Twenty people attended the first meeting, held in September 1980, and they travelled from the genetic services in Cardiff, Edinburgh, Belfast, Nottingham, Manchester, Preston and Cheshire. Eleven were nurses or health visitors, eight were social workers and one was a psychologist (Weetman 1995). Subsequently GNSWA hosted regular meetings around the country where members shared information about different conditions, case histories on specific families and their experiences in managing families.16

In 1988, genetic nurse Sally Farnish reported the results of a survey by GNSWA in the *Journal of Medical Genetics*, under the title, ‘A Developing Role in Genetic Counselling’. Farnish reported that although workers were still widely scattered, often worked in isolation, and had an assortment of job titles, they were now widely recognised as invaluable to the genetic services—especially their skills in establishing crucial relationships with families and clients and in delivering important psychological and material support (Farnish 1988). The growing visibility and autonomy of genetic nurses seems to have occurred in line with calls for nurses in the NHS more generally to be seen less as the ‘doctor’s handmaiden’, and more as a distinct class of professionals for whom ‘care’ was central and visible (Smith 1992, 8–9; McFarlane 1977). Care, psychological awareness and emotional management were new vocabularies for practices of genetic counselling that would take on clearer shape and greater prominence in the 1990s.

### Sickle cell and thalassaemia counselling in the 1980s

In the 1980s, another quite distinct group of nurses and doctors began creating spaces, methods and professional roles to support families and patients affected by a specific class of inherited condition—the ‘haemoglobinopathies’, SCD and thalassaemia. Entirely separate from the regional genetics services, and for many years supported only with short-term funding, sickle cell and thalassaemia clinics defined and employed the UK’s very first professional ‘counsellors’ for inherited conditions. The new clinics developed practices of communication and support tailored to the needs of people experiencing ignorance and racism in the healthcare and welfare services. There are now several rich histories of SCD and thalassaemia in the UK; therefore, here I focus only on what ‘counselling’ meant in these contexts, and who delivered it.17

In the 1960s and 1970s, ignorance of SCD within the NHS meant that testing was sporadic and diagnoses rare.18 For those entering hospital with agonisingly painful crises, there were huge barriers to obtaining effective pain relief. Sufferers asking for strong painkillers were often dismissed by hospital staff as drug addicts. Many healthcare professionals seriously underestimated the severity of the condition, resulting in the death of some patients in hospital waiting rooms.19 Moreover, many patients remained unclear about the implications of the condition's genetic character. One recalled the acute uncertainty they experienced in the early 1970s: ‘[there was] nothing really there to tell me how as a sickle-cell patient—how I could deal with it—how I could live with it—what it meant for marriage and so on—if I had children what were the implications—nothing to tell me about it’ (quoted in Nathoo 2001, 19).

Soon, community activists and a small number of healthcare professionals began advocating for specialist centres to support clients and families with SCD—citing ‘counselling’ as a crucial part of the proposed provision.20 In part building on the work of the London-based charity Organisation for Sickle Cell Research (OSCAR) founded by SCD patient activist Neville Clare, the first sickle cell and thalassaemia centre was established in 1979 at Willesden Hospital, in the north-west London borough of Brent, by Irish-Nigerian nurse health visitor Elizabeth Nneka Anionwu and Yugoslavian-British haematologist Milica Brozović.21 The clinic consisted of two rooms in the Willesden Hospital, where staff ran an open-door blood testing facility in collaboration with the hospital haematology department.22 Soon clinics were also established in Lambeth, Manchester and Liverpool, and by 1985 there were seven across the UK. They were all ad hoc, in the sense that they had been variously established through the collaborative efforts of voluntary organisations, community relations councils, community health councils, individual health visitors, as well as departments of haematology and child health and community medicine (Prashar, Anionwu, and Brozović 1985). For many of these clinics, the only employed member of staff was the ‘sickle cell counsellor’ or ‘thalassaemia counsellor’, who was almost always a qualified nurse health visitor.

Staff at these early clinics had to define this counselling role for the very first time. Anionwu recalls that she had not initially thought it was the clinic’s role to give ‘genetic’ counselling as such, but that her practices were changed by the families she cared for:

We were concerned with the clinical, the nursing (particularly for me) the nursing care, of patients in the hospital. Also, with my health visitor hat on I was concerned to support the families. No, it wasn't *genetic counselling* support at that point. I mean it was to support the family. However, [ it turned out that the families wanted ] the provision of *information* and support. And what were some of the questions they always asked? How did I get it? How come I've only got one child with it? And my other children don't have it? If I have more children, will they get it? Genetic counselling! (Anionwu 2021b)

Anionwu’s broad approach to counselling was in part influenced by the work of sickle cell counsellors in the USA. Finding little advice on SCD in the UK, in the mid-1970s Anionwu travelled to Los Angeles, where she visited one of the sickle cell centres recently established with federal funds (Wailoo 2001, chapter 6; Nelson 2011). She was struck by the very broad support service given to local families, later citing in particular what she had learnt from an African American sickle cell nurse practitioner called Sylvia Lee (Anionwu 2021a, 244). In a return visit to the USA in 1978, Anionwu attended a course on sickle cell counselling at Oakland’s Children’s Hospital Medical Center of Northern California; and she recalled how profoundly useful it was to compare notes with African-American nurses and social workers (Anionwu 1996, 170). Later, she explained: ‘[I] was influenced by what I saw in the States, but also just common sense. You included *everything*’ (Anionwu 2021b).

Counsellors in other sickle cell and thalassaemia clinics took a similar approach. Lola Oni, who started work at the Lambeth clinic in 1982, recalled:

It wasn’t just the genetics. Even if you’re talking to a couple who are at risk of having a child with sickle cell disease … issues will come up that will be to do with their housing problem or this problem or that problem and you link them with social services … you may need to go and do a home visit … [o]r you may need to link with the school, their child’s school. …[Y]es, it is very much a holistic approach in the way in which we work with these families. (Oni 2022)

For Oni and Anionwu, the provision of genetic information was inseparable from broader forms of care and support for individuals and families. This attention to the breadth of counselling encounters also came to include the clinical environment in which encounters happened. Oni argued that the character of the setting was crucially important to the effectiveness of the clinic. Patients themselves, they said, did not want to visit a clinic in a hospital—which for many was associated with the experience of pain and crises—rather, it needed to be in the community, so that, as she put it, the community had ownership of it:

The Lambeth centre was actually in the community … Opposite the bus stop … People used to walk in just to use the loo. And then they would say ‘oh, I’ve always wanted to know about sickle’. ‘Oh, did you? Here’s a leaflet, I’ll talk to you about it. Have you ever been tested? Do you know what your haemoglobin status is?’ ‘No, I don’t’. ‘Do you want to be tested?’ Voila. (Oni 2022)

The experiences of her patients made Oni vividly aware of how much the environment of counselling and support encounters mattered. Indeed, SCD is a genetic condition with a physiological basis, but patients’ experiences of it were defined by widespread ignorance of the condition, lack of treatments, poor understanding of the pain suffered by patients, structural racism, the racism of some healthcare professionals, the lack of funding for specialist care, and the extensive social and political marginalisation of the communities to which many belonged, which in turn affected education, housing and employment. The broad and practical counselling practices developed by those in the clinics were direct responses to these experiences. Although the specialism and holism of these services have remained important and appropriate for these conditions, the historical separation of these services from the mainstream genetics services is emblematic of the way that some groups have been left out of the construction of ‘clinical’ genetics. Nevertheless, in the next section I shall explain how, although SCD and thalassaemia counselling remained separate from that offered in the mainstream genetics services, haemoglobinopathy counsellors identified tacit aspects of encounters that would have a lasting impact on the development of genetic counselling more generally.

### Genetics meets ‘ethnicity’

During the 1980s, Anionwu and other haemoglobinopathy counsellors had begun to pay close attention to the ways in which the so-called ‘ethnic’ identities of clients and families affected their encounters with healthcare professionals.23 In the late 1980s, she and a group of colleagues at the Middlesex Hospital became interested in the outcomes of counselling prior to prenatal diagnosis, and drew attention to the significance of what they called ‘cultural, linguistic, religious, social and economic factors’ (Anionwu *et al*. 1988, 771).24 Anionwu’s attention was also drawn to the social identities of the health professionals themselves. Writing a few years later, she discussed the theme of ‘ethnicity’ in counselling interactions, recounting her own experiences working with patients from the local Gujarati community in Brent (Anionwu 1996).25 Related to the issues of language and religion that she had observed in Brent, Anionwu recalled that the nurses and social workers she had visited in Oakland, California had strongly held the view that ‘culturally appropriate sickle cell services were more suitably offered by black health workers’ (Anionwu 1996, 170).

Anionwu surveyed the experiences of haemoglobinopathy counsellors in clinics around the UK, and found that an overwhelming majority had said that they felt their own ‘ethnic origin’ to be relevant to counselling encounters (Anionwu 1996). Respondents variously commented that matching the ethnic identity of counsellors and patients improved communication, established better report and enabled ‘understand[ing] and respect [of] culture, health beliefs’. One added ‘Many social/psychological and cultural implications of SCD/thalassaemia … colour counselling process[es]’; another wrote, ‘Ethnic origin is always relevant and has an impact on all interactions, needs to be acknowledged, looked at continuously’. Some respondents particularly noted the significance of ‘racism: barriers may exist between white professionals and black and minority clients’ (Anionwu 1996, 181).26

Anionwu’s observations had shifted her attention to the recruitment of staff. By 1985 the Brent clinic obtained NHS funding for two additional counsellors, one of whom was paediatric nurse Nina Patel, herself from India and fluent in Gujarati. In 1986, the Brent clinic produced a poster, that Anionwu hoped would ‘publicise the existence of African, Caribbean and Asian staff’ (Anionwu 1996, 172). The carefully designed poster featured smiling, informal black-and-white portraits of the three counsellors, and prominent positioning of (only) their first names, ‘Marvelle, Elizabeth and Nina’. The poster also showed smaller snapshots of the counsellors interacting with patients, in person or on the telephone. The poster was funded by the Brent Race Relations Unit, and distributed to general practice surgeries, libraries and community centres.

Another health visitor and counsellor, Verna Davis, founded and ran the Manchester Sickle Cell and Thalassemia Centre (established in 1984), and later reflected on the significance of cultural identities in the centre. She recalled, ‘[genetic counselling] is a very emotive subject; it’s one that affects reproductive choices, there are cultural issues, religious issues … and I didn’t think it was adequate trying to interpret through a younger member of the family, a husband’ (quoted in Valier and Bivins 2002). Like Anionwu in Brent, Davis explained, ‘we campaigned vigorously to get an Asian worker involved, a health worker, who could counsel in their own mother tongue’ (quoted in Valier and Bivins 2002). For Davis, fluency of language and cultural understanding were some of the tacit cultural and linguistic dimensions of emotionally charged counselling interactions.

By the early 1990s, some NHS regional genetics centres were starting to follow the lead of sickle cell and haemoglobin clinics and attend to the significance of language, culture and ‘ethnicity’ in genetic counselling. The Yorkshire Regional Genetics Service in Leeds saw that with a large Pakistani population in the city there was an urgent need for professionals who could speak Urdu and Panjabi. In 1992, the Leeds team hired Mushtaq Ahmed who had settled in the UK from Pakistan and had worked in the Leeds Citizen’s Advice Bureau and in mental health services before embarking on his genetic counselling career (Ahmed 2021). Ahmed saw language as crucial (eg, Shaw and Ahmed 2004), and he was aware of the experience and knowledge he brought as a member of the local Pakistani community. Of particular concern was the potential reluctance of families to seek genetic counselling for fear of being blamed for practices of ‘consanguinity’ (that is, marriages between cousins). Such practices were increasingly criticised in UK media, and many in the Pakistani community felt that this important and valued cultural tradition prejudiced them in the eyes of white clinical genetic professionals (Ahmad 1995; Darr 1997).

Indeed, negative perceptions of consanguinity prompted the UK’s nationwide ‘Genetic Interest Group’ to commission a survey and report entitled ‘Access to Genetic Services by Minority Ethnic Populations’ (Darr 1999). Its author, social scientist Aamra Darr, confirmed that many people experienced difficulties in obtaining support and information from professionals who they felt did not respect their cultural background. Ahmed himself worked extensively to teach other professionals how such counselling encounters might best be approached (eg, Ahmed 2013).

In summary, attention to language, ethnicity and culture was one way in which, from the late 1980s and early 1990s, UK healthcare professionals took an interest in the tacit social and cultural conditions of genetic and haemoglobinopathy counselling encounters, which, some practitioners believed profoundly affected what kinds of information could be communicated, how that information was understood and what kind of support was possible. This chimed with intensifying attention on the psychological and social dimensions of such encounters, which I turn to now.

#### Genetics meets the ‘psychosocial’

Several early clinical geneticists had taken an interest in the social contexts and ramifications of counselling. In the early 1970s some had carried out studies to assess the social impact of genetic counselling on families. In the UK and Europe these focused principally on the question of the ‘effectiveness’ of counselling, construed variously as a family’s understanding of disease risk and reoccurrence, or as changes in family composition following genetic counselling.27 Then, increasingly during the 1970s, psychologists and social scientists weighed in on and critiqued the design and methods of some of those earlier studies.28 The first textbook on the psychological aspects of genetic counselling, *Genetic Counselling: A Psychological Approach*, was written by geneticist and psychotherapist Seymour Kessler. There was little mention of this book in the published UK literature until 5 years later when Edinburgh-based clinical geneticists Alan Emery and Ian Pullen edited *Psychological Aspects of Genetic Counselling* (also published by Academic Press). Most of its essays were written by US-based psychiatrists and psychologists, who offered ‘practical guidance to important psychological problems involved in genetic counselling and the skills required to tackle them’.29 Echoing Kessler’s language, Emery and Pullen pointed to what they saw as a turning point away from what they called *‘content-oriented* counselling’ and towards *‘person-oriented* counselling’ (original emphasis) (Emery and Pullen 1984, 4). The editors urged clinicians consider the individual character of genetic counselling encounters, and the multiple psychological and emotional effects that such encounters might have on clients and families. Where UK clinicians had previously framed genetic counselling as being about the communication of accurate genetic information (eg, Harper 1981; Stevenson and Davison 1970), now a new layer was being added to professionals’ understanding of the encounter. As one US-based reviewer affirmed, ‘genetic counselling is at its heart an intensive educational experience that has enormous psychological implications’ (McInerney 1986).

Through these works, UK clinical geneticists likely learnt about research developments in the psychological and emotional ramifications of genetic counselling—although some approached these new themes with trepidation. *Psychological Aspects* was reviewed largely positively in the USA and UK (Connor 1985; Gurling 1987), although a comment in the *Lancet* was particularly striking. There, clinical geneticist, Marcus Pembrey of the Institute of Child Health in London wrote that he ‘felt somewhat threatened when reading it’. He went on to say that he found himself ‘defending the view that genetic counselling services should limit their contribution to what constitutes their special area of expertise, and not try to be all things to all men’, although he added that he had found the book ‘thought-provoking’ and felt it had ‘much to offer’ (Pembrey 1985).

Others, including many nurses, associates and haemoglobinopathy counsellors, engaged with these developments enthusiastically. For many UK workers, their interest in such themes was fostered by connections with the European mainland. Most notably, in the late 1980s and early 1990s, Kerzin-Storrar, Anionwu, genetic nurse Penny Guilbert and genetic counsellor Chris Barnes all attended early meetings of the European Meeting on Psychosocial Aspects of Genetics (EMPAG), an organisation established by a group of social workers in Groningen in the Netherlands (Kerzin-Storrar 2021). Authors of published literature on genetic counselling at the time rarely explicitly defined the term ‘psychosocial’, but used the phrases ‘psychosocial aspects’, or ‘psychosocial dimensions’ of genetic counselling to refer to the range of psychological responses that should be expected in people with different social experiences and identities.30 As well as practitioners, also attending the EMPAG meetings were numerous researchers engaged in flourishing studies on various social and psychological aspects of genetic counselling. Psychologists, sociologists and social scientists presented reports, for example, on communication methods for inviting people to carrier testing, emotional responses to the diagnosis of genetic conditions, methods of conversation analysis, and the roles of emotional responses in reproductive decision-making (eg, “Abstracts of Papers and Posters Presented at the Third European Meeting on Psychosocial Aspects of Genetics” 1993; Barnes, Marteau, and Evers-Kiebooms 1997).

By the early 1990s, some NHS regional genetic centres were engaging explicitly and practically with these innovations. For example, unusually, the Yorkshire Regional Genetics Service in Leeds had on its team a psychotherapist, Christina Oliver, who had trained as a midwife. Anna Middleton, who was associated with the Leeds centre from 1992, recalls the profound influence that Oliver had on the practices of genetic counsellors on the team, and that it was one of the first units in the country to introduce ‘supervision’ for genetic counsellors. For the Leeds team, supervision—by then an established practice in psychotherapy—meant confidential meetings between a counsellor and a colleague in order to reflect on the emotional encounters that they were dealing with, especially with respect to difficult cases.31 It was over a decade before the formal articulation and guidance of what supervision meant in UK genetic counselling (Clarke *et al.* 2007), but Middleton recalled the central role of the practice in Leeds, and how it gave her ‘the chance to learn and see how to deal with emotional issues from a professional standpoint very, very early on’ (Middleton 2022).

The 1990s was the decade of the Human Genome Project and ‘the new genetics’, which attracted attention from disability scholars and activists deeply concerned with the potential for expanded genetic testing to reify a biological determinist concept of disability as medical incapacity.32 Prominent among such critics was sociologist Tom Shakespeare, who wrote extensively about the threat that prenatal screening and selective termination posed for the rights of disabled people (eg, Kerr and Shakespeare 2002; Shakespeare 1995, 1998, 1999). Drawing on comments by Clarke (1991) and Richards (1993), and picking up on research by Michie and Marteau (1996), Shakespeare cautioned that genetic counselling was anything but objective, neutral and ‘non-directive’; and was particularly concerned that genetic counselling ‘may communicate nothing about what it’s like to live with a particular condition’ (Shakespeare 1998, 677).33

During the 1990s, Shakespeare did a great deal of work to engage with those in the clinical genetics services, including teaching on the Manchester MSc in genetic counselling (and he later recalled how his own views also shifted through sustained engagement with Newcastle-based clinical geneticist John Burn, for example).34 Indeed, some of those working in the world of medical genetics had begun to pay close attention to varied ways in which individuals and families with direct experience of genetic conditions might engage with clinical genetics. For example, working in Leeds, Middleton developed a particular interest in encounters between clinical geneticists and people who identify as deaf or as culturally Deaf. Middleton, who is not deaf herself, began this work after being tasked by clinical geneticist Robert Mueller to collect blood from families for his research into the inheritance of deafness. In her paper, published in the *American Journal of Human Genetics* in 1998, Middleton argued that employing deaf genetic counsellors would improve services for deaf people, because of better language communication and because of their cultural awareness: ‘A deaf genetic counsellor would be fluent in sign language and would have a cultural awareness as well as first-hand knowledge of issues relevant to deaf people’ (Middleton, Hewison, and Mueller 1998, 1179). Citing a recent paper by Shakespeare (1998) on the relationship between genetics, eugenics and disability equality, Middleton and her co-authors wrote that the recruitment of deaf counsellors might help to address Shakespeare’s call for greater involvement of disabled people within genetics policy-making (Middleton, Hewison, and Mueller 1998, 1179).

I have described here how, in the mid-to-late 1980s and 1990s, UK healthcare professionals began to reinvent genetic counselling as a psychological, emotional and social encounter. Genetic nurses, social workers and haemoglobinopathy counsellors, in collaboration with clinical geneticists, sociologists, psychologists and other social scientists, created forums for exchanging ideas and practices, a flourishing vocabulary, and an increasingly varied body of research on the social and psychological responses of clients and families to clinical genetic information. It was this ‘psychosocial’ emphasis that would become a central feature of the new genetic counselling master’s course first offered in Manchester in 1992.

### Making the genetic counsellor

As we have seen so far, the 1980s was formative for the UK’s development of practices delivered in regional genetics centres by genetic nurses, and within the haemoglobinopathy services by sickle cell counsellors and thalassaemia counsellors. During the early part of that decade, those ‘non-medical’ counselling roles were still relatively invisible—in the sense that they did not involve nationally organised training and they were rarely mentioned in journals or textbooks. There were also few opportunities for formal training, which tended to happen ‘in-house’ within individual institutions. One exception was a week-long training course for haemoglobinopathy counsellors run by Elizabeth Anionwu at the Brent Sickle Cell and Thalassaemia Clinic (Anionwu 2021b). Another was a week-long residential training course established in 1989 in Cardiff, developed by genetic nurse Mary Rogers in collaboration with colleagues within GNSWA, and accredited by the Welsh Nursing Board (Reynolds and Tansey 2010). Then in the early 1990s, Lauren Kerzin-Storrar, a US-trained genetic associate working in Manchester, took the first steps towards establishing the UK’s first masters-level training course in genetic counselling. She was supported in this endeavour by clinical geneticists Rodney Harris and Dian Donnai. In doing so, Kerzin-Storrar would introduce into the community a range of methods developed at Berkeley, California, including its emphasis on the psychosocial elements of counselling.

Born in the USA, Kerzin-Storrar had been studying in San Diego for a first degree in biology in the mid-1970s when she heard about the master’s course in Genetic Counselling at Berkeley, California. The first such graduate degree had been established at Sarah Lawrence College in New York in 1969; a handful of others followed suit and the Berkeley course was set up in 1973.35 That programme was part of a new initiative at Berkeley to promote community and integrative health, and a founding tenet of the programme was an emphasis on collective health and holistic healing. Thus, students on the course learnt not just about the science of genetics and the clinical care of inherited conditions, but were expected to gain insights into the social experience of disability or reproductive decision-making from placements in a range of settings, which included special schools and family planning clinics. The course was directed by Kessler, who promoted a vision of genetic counselling as social-psychological communication. He and the other organisers strongly emphasised a focus on psychological theories and strategies (Stern 2012). In this model, genetic counsellors were understood as part of a psychological interaction, which aimed to acknowledge the emotional impact for clients of the information being discussed, and to facilitate personally meaningful decision-making. Kessler emphasised techniques of audio recording and role-playing, which he believed (as Stern recounts) gave students a deeper understanding of the counsellor and counselled ‘as human beings working through what likely were painful and difficult feelings related to genetic disease and disability’ (Stern 2012, 140).36

The orientation of the Berkeley degree had a profound impact on Kerzin-Storrar’s own vision and practice, and on her establishment of the Manchester masters in genetic counselling in the early 1990s.37 Like the Berkeley course, Kerzin-Storrar arranged the Manchester masters in three parts, with taught modules (lectures, seminars, essays, on eg, clinical genetics, ethics, epidemiology and statistics), placements (the first in non-genetic healthcare, community and social service setting, the second in a clinical genetics unit) and a research project. Students were schooled in the practical techniques of understanding, interpreting and communicating with clients, using recording and videotape of role-play and real sessions with clients’ consent.38 One taught module, ‘Counselling and Care in Health Settings’, focused on the meanings and lived experience of disability and illness, and which Kerzin-Storrar modelled on a Berkeley first year module taught by social worker Judith Tiktinsky.39

Also teaching on the course was Elizabeth Anionwu, by now Lecturer in Community Genetics at the Institute of Child Health at UCL. Anionwu schooled students on haemoglobin-related conditions, but also on issues of ‘ethnicity’ and counselling interactions (discussed in the previous section). Recalling Anionwu’s insights into social identity and counselling, Kerzin-Storrar commented, ‘frankly in those early days that would have been the only…thing in the entire course that actually addressed [issues of culture and identity] directly’ (Kerzin-Storrar 2022). Anionwu’s contribution to the course was one instance of communication across the disciplinary divide between ‘haemoglobin’ and ‘genetics’ services—then still a rarity noted by the aforementioned Genetic Interest Group report.40

Like in Berkeley, the Manchester degree put students on placements in social care, educational or health settings, which they would do with supervision, to introduce them to the experience of reflective practice (Kerzin-Storrar 2022). Organisers saw these placements as, in part, a way of bridging the expertise of those coming from nursing training (who already had extensive experience of such settings) and those with science degrees. In creating the course, and by extension the new identity of the ‘genetic counsellor’, the Manchester group consciously attempted to bring into alignment students with expertise in nursing and social work and those with science degrees.

This alignment work happened in concert with another institutional change. In 1995, the GNSWA voted to change its name to the Association of Genetic Nurses and Counsellors (the AGNC); a letter circulated to services around the country explained, ‘it was felt that this [name] best describes the focus of most of the membership’.41 The AGNC also started creating a registration process for genetic counsellors, with the aim of providing a benchmark for skills and expertise. The organisation’s new name was controversial—‘social workers’ were no longer in the title, and it affirmed the visibility of the identity of the ‘genetic counsellor’. Some remember unhappiness among the membership; one (former nurse) recalled, ‘I think [that some nurses] very much felt that they were being left behind … In a way it was changing the future of their whole profession’ (Barnes 2021). So, for example, in creating the registration process, the AGNC leadership (many of whom were genetic nurses themselves) incorporated into the system a ‘grandparenting clause’, which stipulated that genetic nurses could apply for registration on the basis of their nursing qualifications and a portfolio drawn from their practical experience. Organisers felt that this acknowledged that a lot of the experienced nurses should not be expected to devote the time and expense to completing a master’s degree.

As well as the delicate knitting together of different specialities, Kerzin-Storrar recalls the work involved in creating a space for MSc-qualified counsellors within the national professional landscape (Kerzin-Storrar 2021; 2022). Some clinical geneticists in Britain remained sceptical about the value of the new degree. Many still believed that genetic counselling should be done only by clinical geneticists, and at the same time as discussions about diagnosis and family history. Although personally supportive of Kerzin-Storrar, Peter Harper was one such sceptic. The fourth edition of *Practical Genetic Counselling* (1993) explained that, ‘genetic counselling should preferably be undertaken by people who are medically trained, largely for the reason that it is quite impossible to separate the actual counselling from the associated aspects of clinical diagnosis’ (Harper 1993, 140). He had misgivings about what he saw as ‘US-style’ genetic counsellor, owing to the apparent animosity that had developed between some professional groups in the USA (Stern 2012; Stillwell 2015).42

Harper was by no means alone in his concerns; many regional centres were resistant to the new degree and wary of employing its graduates. Today Kerzin-Storrar perceives that resistance to have been partly structural—ultimately, genetic counsellors were limited in their autonomy because the NHS services worked in teams, and the consultants in those teams took responsibility for cases (Kerzin-Storrar 2022). Kerzin-Storrar and her colleagues worked to persuade centres that masters-trained genetic counsellors were qualified and desirable. Every year course organisers brought in a couple of students who were seconded from one of the regional genetics centres, as a strategy for embedding the service in the course and communicating the qualities of the course to their colleagues. Eventually they crossed a threshold and there was a critical mass of qualified counsellors working in centres around the country.

By 1995, genetic counsellors were an accepted part of the NHS clinical genetics community. The role of the ‘genetic counsellor’ had not replaced that of the genetic nurse, but represented a more formal articulation of a role first developed by nurses and now practised by people with different training backgrounds. In 1995, the newly named AGNC was brought under the umbrella of the new ‘British Society for Human Genetics’—an organisation that also included the Clinical Genetics Society, the Association for Clinical Cytogenetics and the Clinical Molecular Genetics Society. This served to underline the successful integration of genetic counsellors with nurses, and signal their mutually visible role within the broader field. And significant for many of those in the AGNC, it brought the genetic counsellors into the new society on an equal footing with clinical geneticists.

## Conclusion

It is something of a coincidence that both haemoglobinopathy counselling and genetic counselling in the UK were (separately) influenced by developments on the US West Coast. The work of Elizabeth Anionwu and her colleagues in Brent was modelled on the broad support given by sickle cell counsellors in Los Angeles and Oakland—who also influenced growing interest in and attention to notions of culture and identity in counselling encounters. Meanwhile, Lauren Kerzin-Storrar brought to the Manchester degree several features of the Berkeley genetic counselling course, including an emphasis on the value of community placements, on psychological theories and issues, and on reflexive practice. Although the UK’s first master’s courses in genetic counselling lagged the US by over two decades, this perceived ‘delay’ does more to reveal some of the distinctive features of clinical genetics in the NHS. For example, in contrast to the US landscape of private healthcare, the context of state-run, nationwide, regionally organised health services—characterised by cooperation within teams and attempts to standardise services between regions—likely slowed changes to the professional identities of nurses, counsellors and social workers. The ‘team’ character of regional genetics centres already created the conditions for a local diversity of roles, training and expertise, which encouraged but also absorbed change. It is also likely that the NHS administrative structure tempered the pace of change compared with the USA. For example, Kerzin-Storrar noted that for many years her hospital Chief Executive, who supported her original appointment, did not know what to pay her, because she did not fit into an existing salary scale.43

Within the NHS clinical genetics services, we have seen the growing significance of the emotional labour of genetic counselling. The emotional management of clients and families by ‘auxiliary workers’ was largely unacknowledged in print until the late 1980s, perhaps both cause and consequence of this work being done by women.44 Indeed, the growing formality of emotional, psychological and social practices in clinical genetics broadly parallels the expanding visibility of care and emotional management by nurses more generally in Britain. Perhaps most famously, Pam Smith’s influential study (Smith 1992) sought to transform some of the relatively invisible dimensions of nursing care into practices that could be articulated, theorised and taught. But although ‘(in)visibility’ is one lens through which we might view this history, we have also seen how practitioners and researchers created new ideas and practices, a flourishing vocabulary, and an increasingly varied range of experiences of the social and psychological responses of clients and families. In this sense, genetic nurses, social workers and haemoglobinopathy counsellors, in collaboration with clinical geneticists, sociologists, psychologists and other social scientists, made, or reinvented, genetic counselling as a psychological, emotional and social encounter.

Perhaps because of the commitment to team working within the NHS genetic services, the history of genetic counselling in the UK draws attention to a great deal of careful negotiation and management of roles and identities across the system of clinical genetics. Devon Stillwell, in her account of genetic counselling in the US, draws on Andrew Abbott’s concept of an interrelated ‘system of professions’—in which the development of one profession is shaped by competition with others it is in contact with—to describe the complicated gender culture that structured genetic counselling (Abbott 1988; Stillwell 2015). Stillwell’s focus was on a sometimes-combative relationship between genetic counsellors and physicians (see also Stern 2012). In the story I describe, we see an even more complex negotiation within the system of NHS clinical genetics—with those advocating for the identity of the ‘genetic counsellor’ negotiating both with clinical genetics *and* with genetic nurses and social workers to carve out their new roles and expertise.

The dynamics of the relationships between UK genetic nurses and counsellors, on the one hand, and clinical geneticists, on the other, was complex and varied. Some former nurses described the paternalistic attitudes of clinical geneticists and other specialists. Others chose to emphasise the extensive support and autonomy they were given by the clinicians they worked with. Genetic nurse Heather Skirton explained, ‘I always used to think how it happened in this country was a few medical geneticists started saying, ‘my nurse can do more than just change the bedsheet on the examination table and make my coffee’’. She reflected on how those relationships likely affected the trajectory and pace of change of particular services (Skirton 2021). The contrasting recollections of such relationships in different places reflects the varied ways in which genetic nurses and counsellors negotiated the constraints and opportunities of their field. Further work will explore how these relations map onto other feminist and antiracist efforts to change the experience and expertise of NHS healthcare.

I have described how the expanding autonomy of genetic nurses and the construction of the genetic counsellor developed in concert with a turn to the ‘psychosocial’ in clinical genetics. We might understand this turn as part of a conscious distancing from ‘eugenics’, a shift that was then itself complicated by debates in the late 1980s and early 1990s about ‘non-directive counselling’.45 It is intriguing that for academic psychologists, the notion of the ‘psychosocial’ had lost its allure postwar in the face of efforts by evolutionary psychologists and ethologists to redescribe social relationships in biological terms (Hayward 2012). And yet, evidently, in the 1980s, the term was given a new purpose by those in clinical genetics. Further work will explore how the concept of the psychosocial functioned to deflect and counter the field’s historical relationship to eugenics, and now, in concert, it became a way of expressing how genetics could be made meaningful to the varied social and psychological experiences of people.

This history is ongoing. As genomics becomes ever more visible within UK healthcare, genetic counsellors continue to carve out roles as essential mediators and interpreters of such data.46 In response to the expanding claims for genomics within the NHS, representatives of the AGNC recently published an updated description of the roles and scope of genetic counsellors in the UK, in which the authors explained that ‘genetic counsellors are playing a key role in enabling non-genetic health professionals learn, understand and integrate genomic data into their practice’ (Middleton et al. 2023). Tracing the historical and ongoing dynamics of expertise around the interpretation and communication of genetic data is crucial for understanding how genetics has been, and continues to be, made meaningful and valuable to people, families and their communities.

## Data Availability

No data are available.

## Notes

1. 1. In line with present-day practice, I use the term ‘client’ to refer to a person engaged in genetic counselling with a professional (Stern 2012); in the place and period about I am writing, clients were sometimes referred to as ‘patients’, a term I occasionally use here, especially with reference to the sickle cell and thalassaemia services.

2. 2. Few accounts touch on the history of genetic counselling in the UK; exceptions include Coventry and Pickstone (1999); two Wellcome Witness Seminars (Reynolds and Tansey 2010; Jones and Tansey 2014) and clinical geneticist Peter Harper’s recent historical compendium (Harper 2020). There is growing scholarship on the history of genetic counselling in Canada, France, Mexico, Russia, Greece, Cyprus, East and West Germany, Belgium and Sweden (Barahona 2017; Baranov 2017; Barmpouti 2017; Björkman and Moser 2017; Cowan 2009; Doetz 2017; Gaudillière 2011; Leeming 2004; Nemec and Moser 2017; Vandendriessche 2017).

3. 3. The visibility of care and emotional management expanded in concert with attention to the ‘emotional labour’ of nursing, articulated by James (1989) and Smith (1992). Tierney, Bivins, and Seers 2019 observe the emergence in the 1980s of a discourse around ‘compassion’ in nursing in the context of an increasingly marketised NHS.

4. 4. Many early UK genetics clinics were established by researchers without medical degrees, or by medics who were not geneticists (Harper 2020).

5. 5. This resonates with an account of another gendered professional identity in the NHS: the medical social worker (Gosling 2018).

6. 6. On the continued inadequacy of sickle cell patient care in the NHS today, see Mahase (2021).

7. 7. This cohered with late 1980s’ and 1990s’ interest in the multiplicity of embodied subjectivities and theoretical critiques of the ‘universal’ psychological self: for example, Haraway (1989); Butler (1993).

8. 8. This connects to new scholarship on the management of encounters with patients, eg, Hayward (2014), Hitzer (2022), Greene (2022), and Brown (2023).

9. 9. I use the verb ‘to make’ in order emphasise the active, practical work of creating genetic counselling encounters (for a useful review of the ‘turn to practice’ in the history of science, see the introduction to Soler *et al*. (2017)). I use the term ‘to reinvent’ to resonate with argument that the formation of new vocabularies, explanations and forms of expertise within the ‘psy’ sciences effectively created new ways in which people came to understand themselves (Rose 1996). Both terms help to avoid implying that the changes to professional genetic counselling encounters were inevitable.

10. 10. Patients and/or the public were not involved in the design, or conduct, or reporting, or dissemination plans of this research. The recruitment, planning, conduct and data storage of these interviews were approved by the Queen Mary Ethics of Research Committee (QMERC20.157). I selected interviewees through their published contributions and through chain referral. At first contact (usually by email), interviewees were given my approved ‘Participant Information Sheet’ and consent form before deciding whether they wanted to participate. My Participant Information Sheet described how the data resulting from the interview would be stored and used, and about my data protection and privacy policies, which conformed to standards set by the Queen Mary Ethics of Research Committee. Both my recruitment email and sample interview schedule were approved by the Queen Mary Ethics of Research Committee. I devised by interview schedule in relation to guidance from the Oral History Society and the 5 day British Library/Wellcome Oral History training course (2011); and using guidance from three short qualitative interviewing courses, at Goldsmiths College (2015), at the Social Research Association (2015); and the Patient Experiences Research Group at the University of Oxford (2022). In 20 interviews carried out between January 2021 and November 2022, my questions were informed by the historical trajectories that I already knew and developed iteratively as I interviewed more people. After covering the interviewees’ early life and education, the main portion of each interview concerned their professional training and career, and so questions were specific to the person and their professional history. Transcription was carried out by me or by a professional transcriber, who conformed to the privacy guidance required by the Queen Mary Ethics of Research Committee. I coded the interviews iteratively using the Nvivo software.

11. 11. The first NHS clinical training posts in genetic medicine were created in 1978 and formed the basis for a new career structure in medical genetics. The regional genetic centres varied in size—more established centres were closely associated with an academic medical genetics unit; newer centres were smaller and wholly clinically oriented (Harper 2020, 190). All had ties with local hospital cytogenetic and other laboratory services. Services were patchy—in 1982 the Clinical Genetics Society found that at least eight regions of the UK had no NHS-funded consultant, nine had no nursing staff, and most had inadequate administrative staff (Fitzsimmons et al. 1982).

12. 12. Several other women worked in Carter's MRC unit, who primarily collected family data for research (Reynolds and Tansey 2010, 29). In another example of underrepresented female labour in this field: at the UK’s earliest genetic counselling clinic at Great Ormond Street Hospital, Margaret Fraser Roberts worked as her husband John’s personal assistant and computer, assisting him in clinics, making notes on cases prior to clinics, and creating brief précis about each family or patient (Reynolds and Tansey 2010, 29).

13. 13. The phrase ‘field worker’ may have been an allusion to the long-standing 'field' practices in human genetics of recruiting and liaising with research subjects (Comfort 2012, 40).

14. 14. Harper’s declaration was in part aimed at non-medically trained geneticists who might be tempted to attempt counselling; see also Stevenson and Davison (1970, 306).

15. 15. For example, the Clinical Genetics Society report ‘The Provision of Regional Genetic Services in the United Kingdom’ (Fitzsimmons et al. 1982, 11–12).

16. 16. At these meetings, participants learnt that there was significant variation around the country as to the autonomy granted to genetic nurses in relation to other team members.

17. 17. On SCD in the UK: Nathoo (2001); Bivins (2015), chapter 6; Bivins (2017); Redhead 2019; autobiographical accounts of SCD in the UK include Clare (2007); Anionwu (2016, 2021a).

18. 18. Historian Roberta Bivins describes in detail inaction on SCD by the Ministry of Health and the deep politicisation and racialisation of the health of immigrant populations postwar [Bibr R21].

19. 19. Nathoo (2001). For patient experiences, see Redhead (2021); Anionwu (2021a).

20. 20. For example, the political magazine *Race Today* called for a nationwide plan for sickle cell action, which would include ‘counselling units…established in hospitals around the country’ (Crawford 1974); the *British Medical Journal* published an editorial calling for clinics local to ‘areas with a large immigrant population’ to provide ‘[f]aculties for family investigations and for genetic counselling’ ([Bibr R94]).

21. 21. For more on OSCAR, including personal testimonies from Clare and others: Nathoo (2001); and Clare’s autobiography (Clare 2007).

22. 22. Anionwu and others founded the Sickle Cell Society, which created and published numerous reports documenting nationwide needs and provision (eg, Sickle Cell Disease: The Need for Improved Services 1983). On the involvement of haemotology clinicians and laboratory staff in some sickle cell and thalassaemia centres, see Anionwu 2016.

23. 23. The term ‘ethnicity’ had varied meanings in healthcare at this time, for example, Kelleher (1996), Darr (1997), Macbeth (1997) and Sharma (1997).

24. 24. The 1980s and 1990s saw the publication of several reports and analyses of experiences by minority communities of UK healthcare (eg, Ahmad 1994; Beishon, Virdee, and Hagell 1995; Hillier and Kelleher 1996; Scrivens and Hillier 1982).

25. 25. Gujarat is a state of north-western India, adjacent to Pakistan.

26. 26. Not everyone agreed that ‘ethnicity’ mattered. Lola Oni, who worked at the clinics in Lambeth and then Brent, observed that people labelled with the same ‘ethnic’ identity might have vastly different cultural attitudes. From running training programmes in Nigeria Oni found that people from different groups could have very different attitudes to inherited conditions; and observed that London-based Caribbean and African parents had different attitudes to having a child with sickle cell disease (Oni 2007). She also noted that the supposed matching of social identities could sometimes preclude effective counselling, and observed that the small size of the healthcare workforce often made cultural matching impractical (Oni 2022).

27. 27. For UK examples, see: Carter *et al.* (1971); Emery, Watt, and Clack (1973); McCrae *et al.* (1973); Pearn (1973). Various US contributions on similar themes were published in Lubs and de la Cruz (1977).

28. 28. See, for example, the work of prominent analyst in Europe, Belgian psychologist Gerry Evers-Kiebooms (eg, Evers-Kiebooms and van den Berghe 1979).

29. 29. Emery and Pullen (1984) (cover blurb).

30. 30. For example, Kessler (1979, ‘Preface’); historian Rhodri Hayward explains that, historically, the concept of the ‘psychosocial’ was ‘both ill defined and over-determined’; he traces its uses by academic and later clinical psychologists in the late nineteenth and twentieth centuries (Hayward 2012).

31. 31. Much literature on supervision in psychotherapy was published in the USA, although contemporary textbooks extensively reviewed in the UK include Hess (1980).

32. 32. The phrase ‘the new genetics’ was used to refer to the rapid expansion of knowledge about the genetic mechanisms of disease conditions and availability of genetic testing; for example, Bell (1998).

33. 33. On non-directive genetic counselling in the USA, Stern (2012).

34. 34. On Shakespeare’s involvement on the MSc, Kerzin-Storrar (2022); for autobiographical reflections on his work in the 1990s, Shakespeare (2019).

35. 35. On the expansion of masters courses in genetic counselling in the USA, see Stern (2012).

36. 36. Kerzin-Storrar herself recalled from her student days taping actor-led consultations and participating in interviews, which she remembered as ‘really integral alongside all the genetics and clinical genetics we were being taught’ (Kerzin-Storrar 2021).

37. 37. Several of Kerzin-Storrar’s colleauges were key to implementing the master’s programme: among them, David Craufurd was a psychiatrist specialising in inherited neuropsychiatric disorders, and Tara Clancy and Rhona Macleod were recently appointed masters-level nurses. Kerzin-Storrar, personal communication with the author, 2022.

38. 38. One former student recalls teaching from an oncologist on the practices of ‘breaking bad news’ (Middleton, 12 May 2022); historian Bettina Hitzer has analysed changing talking practices in oncology (Hitzer 2022, chapter 4)

39. 39. Lauren Kerzin-Storrar, private collection, LKS_3, c. 1977.

40. 40. The report concluded that, ‘the lessons learnt [by haemoglobinopathy services], through research and experience about service delivery to linguistically and culturally diverse minority ethnic populations, have not filtered through into RGC [Regional Genetics Centre] practice’ (Darr 1999, 7).

41. 41. The letter was authored by Chairman of the AGNC, genetic nurse Penny Guilbert (Guilbert 1995).

42. 42. Harper later was strongly in favour of genetic counsellors, and supported the establishment of the second UK master’s course in Cardiff, which was modelled on the Manchester course.

43. 43. Kerzin-Storrar 2022.

44. 44. On the relationship between gender and visibility in professional work, for example, Hicks (2017) and Egginton and Thomas (2021).

45. 45. ‘Eugenics’ (a word with varied and changing meanings) is not a history I have addressed here directly, but for the strategies used to distance genetics from ‘eugenics’, see Ramsden (2009); and for the public debate in the early 1990s about the ethics and logic of testing in the context of nationalised healthcare (eg, Clarke 1991; Super *et al*. 1991).

46. 46. For example, the government recently announced a new venture to explore the effectiveness of whole-genome sequencing in newborn babies (eg, Over £175 Million for Cutting-Edge Genomics Research 2022).
